# Comparison of the feasibility and validity of a one-level and a two-level erector spinae plane block combined with general anesthesia for patients undergoing lumbar surgery

**DOI:** 10.3389/fsurg.2022.1020273

**Published:** 2023-01-06

**Authors:** Shaoqiang Zheng, Yan Zhou, Wenchao Zhang, Yaoping Zhao, Lin Hu, Shan Zheng, Geng Wang, Tianlong Wang

**Affiliations:** ^1^ Department of Anesthesiology, Beijing Jishuitan Hospital, Beijing, China; ^2^ Department of Spinal Surgery, Beijng Jishuitan Hospital, Beijing, China; ^3^ Beijing Institute of Traumatology and Orthopaedics, Beijing, China; ^4^ Department of Anesthesiology, Xuanwu Hospital, Capital Medical University, Beijing, China

**Keywords:** erector spinae plane block, one-level and two-level ESP block, pain sensorial blockage, lumbar surgery, perioperative analgesia

## Abstract

**Background:**

Spinal surgery causes severe postoperative pain. An erector spinae plane (ESP) block can relieve postoperative pain, but the optimal blocking method has not been defined. The aim of this study is to compare the feasibility of a one-level and a two-level lumbar ESP block and their effect on intraoperative and postoperative analgesia in lumbar spinal surgery.

**Methods:**

A total of 83 adult patients who were scheduled for posterior lumbar interbody fusion were randomly divided into two groups. Patients in Group I (*n* = 42) received an ultrasound-guided bilateral one-level ESP block with 0.3% ropivacaine, while patients in Group II (*n* = 41) received a bilateral two-level ESP block. Blocking effectiveness was evaluated, including whether a sensory block covered the surgical incision, sensory decrease in anterior thigh, and quadriceps strength decrease. Intraoperative anesthetic dosage, postoperative visual analogue scale scores of pain, opioid consumption, rescue analgesia, and opioid-related side effects were analyzed.

**Results:**

Of the total number, 80 patients completed the clinical trial and were included in the analysis, with 40 in each group. The time to complete the ESP block was significantly longer in Group II than in Group I (16.0 [14.3, 17.0] min vs. 9.0 [8.3, 9.0] min, *P *= 0.000). The rate of the sensory block covering the surgical incision at 30 min was significantly higher in Group II than in Group I (100% [40/40] vs. 85.0% [34/40], *P *= 0.026). The rate of the sensory block in the anterior thigh was higher in Group II (43.8% [35/80] vs. 27.5% [22/80], *P *= 0.032), but the rate of quadriceps strength decrease did not differ significantly between the groups. The mean effect–site remifentanil concentration during intervertebral decompression was lower in Group II than in Group I (2.9 ± 0.3 ng/ml vs. 3.3 ± 0.5 ng/ml, *P *= 0.007).There were no significant differences between the groups in terms of intraoperative analgesic consumption, postoperative analgesic consumption, and postoperative VAS pain scores at rest and with movement within 24 h. There were no block failures, block-related complications, and postoperative infection.

**Conclusions:**

Among patients undergoing posterior lumbar interbody fusion, the two-level ESP block provided a higher rate of coverage of the surgical incision by the sensory block when compared with the one-level method, without increasing the incidence of procedure-related complications.

**Clinical Trial Registration:**

www.chictr.org.cn, identifier: ChiCTR2100043596

## Introduction

Posterior lumbar surgery is a common procedure to treat lumbar degenerative diseases (1). The surgery is traumatic and causes postoperative pain, which confines patients to the bed at the early stage, resulting in delayed recovery, prolonged hospital stays, and increased costs (2–4). Perioperative pain management is important for achieving both anesthesia and surgical outcomes (5, 6).

Multimodal analgesia includes intravenous opioids, local anesthetic infiltration, and regional nerve blocking. NSAIDS has also been used in spinal surgery (7). An erector spinae plane (ESP) block is a paraspinal fascial plane block, first reported by Forero et al. in 2016 (8). Local anesthetic (LA) is administered between the thoracic transverse processes and the erector spinae muscle, blocking the dorsal and ventral rami of the thoracic and abdominal spinal nerves (8–10). It has been reported that the ESP block can provide analgesia for lumbar spinal surgery and has opioid-sparing effects (5, 11, 12). Different ESP block methods have been used in previous studies, with different concentrations (0.25%–0.4%) and volumes of bupivacaine or ropivacaine at T10, T12, and L4 or the midpoint of the incision (5, 7, 12–15). There is no systematic evaluation for determining the effects of a lumbar ESP block. It is not clear whether different volumes and injection sites will lead to different outcomes in a lumbar ESP block. We hypothesized that a two-level ESP block would have a higher rate of coverage of the surgical incision by the sensory block compared with a one-level ESP block.

The purpose of this study was to compare the feasibility of the one-level and two-level lumbar ESP block and their effect on intraoperative and postoperative analgesia in lumbar spinal surgery.

## Materials and methods

### Patients

This study was a randomized controlled trial conducted in Beijing Jishuitan Hospital. The study was approved by the Ethics Committee of Beijing Jishuitan Hospital (Review No. 20191202-J02) and was registered at the Chinese Clinical Trial Registry (ChiCTR2100043596). Written informed consent was obtained from all participants.

The patients’ inclusion criteria were the American Society of Anesthesiologists (ASA) physical status class I or II, age ≥18 years, and those scheduled for posterior lumbar interbody fusion. Exclusion criteria included severe heart, kidneys, liver, and life-threatening hematologic diseases; central or peripheral neurologic disease; non-sinus heart rate; pacemaker or antiarrhythmic drug use; allergy to amide-type local anesthetics; infection in the intervention region; a history of lumbar surgery and consuming narcotic substances or alcohol dependence.

Random allocation was performed by using SPSS software Version 22.0 (IBM Corp., Armonk, NY, United States). The inclusion orders 1–80 were inputted, the corresponding random numbers were generated by “COMPUTE random = RV.UNIFORM(0,1),” 40 smaller numbers were assigned to Group I, and the rest were included in Group II. The anesthesiologist who performed the ESP block was given an envelope containing group information when a patient was included. The patients were not informed of their group assignments. Orthopedists were unware of their group assignments. General anesthesia and postoperative assessment was performed by researchers blinded to the group assignment.

### Anesthesia management

#### Conduct of the ESP block

The patients were transferred to the regional anesthesia room 45 min before surgery. A standard monitor was established with pulse oximetry, non-invasive arterial blood pressure measurement, and electrocardiogram. The patients were placed in the right lateral position and given intravenous midazolam of 2 mg and sufentanil of 5 µg for preprocedure sedation.

The ultrasound probe was placed at the sagittal axis, scanning from 5 cm to the midline and moving toward the midline. In sequence, the vertebral transverse process, lamina, and the spinous process were seen. Vertebral level T_12_ could be identified by the 12th rib, followed by each lumbar process. L_1_–L_5_ lumbar spinous processes were marked on the skin to identify operative vertebrae. The objective site of injection was decided according to the surgical incision. The upper site was one vertebral above the operative vertebrae. The lower site was defined as the lowest operative vertebrae. For patients in Group I, the ESP block was performed only at the upper level. For patients in Group II, it was performed at both upper and lower levels. In Group I, each patient received bilateral blocking at the upper level, while in Group II, each received bilateral upper- and lower-level injections. As a result, there were 80 injections in Group I and 160 injections in Group II. The ESP block was performed by the same anesthesiologist.

Ultrasound probe and the region scheduled for the procedure were sterilized. The probe was installed along the sagittal axis at the midline of the targeted vertebral level. The spinous processes were first visualized, and then with the probe moving to the lateral side, the transverse processes and the erector spinae muscle were visualized approximately 3–4 cm to the midline. A 100 mm needle was inserted using the in-plane technique. The needle was targeted between the transverse process and the deep fascia of the erector spinae muscle. The location of the needle was confirmed with 2 ml saline solution, followed by 0.3% ropivacaine injection. The same procedure was also performed on the opposite side. At the upper level, the needle was inserted from the cranial side to the caudal side, and 0.3% local anesthetic of 25 ml was injected. At the lower level, the needle was inserted from the caudal to the cranial side, and the LA volume was set at 10 ml. Therefore, the total LA volume was 35 in Group II and 25 ml in Group I.

#### General anesthesia and operation

After the patients arrived in the operating room, pulse oximetry, invasive arterial blood pressure measurement, electrocardiogram, Bispectral Index (BIS), and Pain Rating Index (Pti) monitor were established. General anesthesia was performed in all patients with a target-controlled infusion (TCI) of propofol and remifentanil. The initial plasma concentration of propofol was set at 3.5 µg/ml and increased by 0.3 µg/ml gradually until eyelash reflex disappeared. When the BIS was lower than 60, the effect compartment concentration was recorded. When the effect compartment concentration of remifentanil reached 3.0 ng/ml, rocuronium of 0.6 mg/kg was injected, and then endotracheal intubation was performed. Propofol and remifentanil were titrated to keep the BIS at 40–60 and PTI at 40–79. If it was necessary, rocuronium of 0.2 mg/kg would be added for muscle relaxation. During the intraoperative period, vasoactive drugs were administrated to maintain the heart rate and blood pressure within 20% of baseline. The operation was performed by the same surgical team using the same technique for all patients.

At 30 min before the end of surgery, parecoxib sodium of 40 mg and tropisetron hydrochloride of 5 mg were injected intravenously. After 15 min, sufentanil of 0.1 µg/kg was administered. The total dosage of intraoperative propofol, remifentanil, and rocuronium were recorded. Postoperatively, neostigmine of 2 mg and atropine of 1 mg were administered to antagonize the residual muscle relaxation. The patients were extubated after all extubation criteria were met and then transferred to the postanesthesia care unit (PACU). Patients with Aldrete scores ≥9 were transferred to the surgical ward.

#### Postoperative analgesia

Patients in both groups were provided with the same postoperative analgesia. Parecoxib sodium of 40 mg was given every 12 h within 72 h after surgery. The protocol of the patient-controlled analgesia (PCA) devices was set with sufentanil of 180 µg, tropisetron hydrochloride of 15 mg, and normal saline of 120 ml and initiated at the PACU. The PCA parameters were set as a basal infusion of 1 ml/h, lockout interval of 30 min, and bolus of 2 ml. For rescue analgesia, tramadol of 100 mg was intravenously administered to patients with visual analog scale (VAS) scores of more than 4 at rest.

### Data collection

General information of the patients was recorded, such as sex, age, height, weight, ASA classification, and duration of surgery. The objective vertebra and time to complete the block were also recorded.

**Figure 1:**
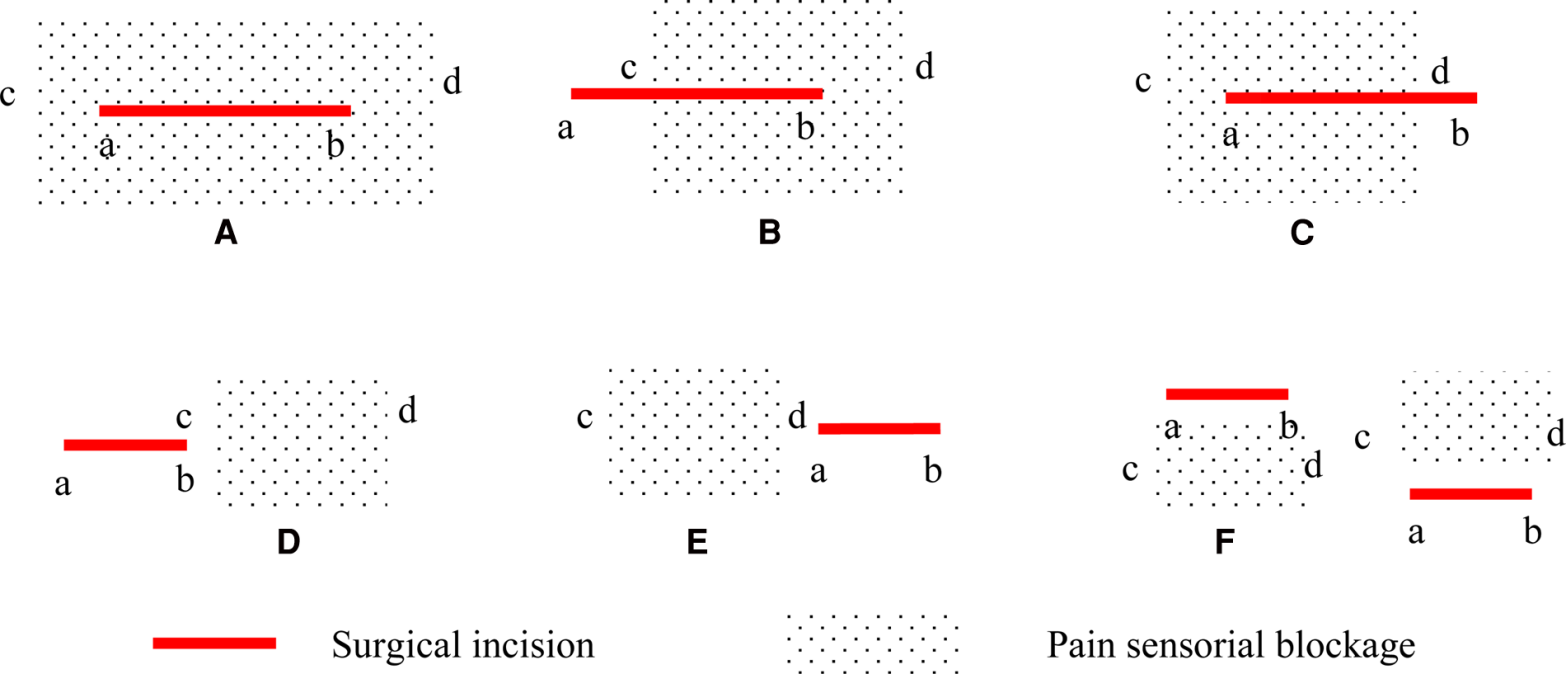
Surgical incision and pain sensorial blockage. (**A**) the coverage rate is defined as complete blocking (100%); (**B**) the coverage rate is defined as cb/ab * 100%; (**C**) the coverage rate is defined as ad/ab * 100%; (**D**) or (**E**) the coverage rate is defined as 0%; (**F**) or no hypoalgesia: blocking failure. a, the upper bound of the surgical incision; b, the lower bound of the surgical incision; c, the upper bound of pain sensorial blockage; d, the lower bound of pain sensorial blockage.

The primary endpoint was the rate of complete coverage of the surgical incision site by the sensory block. The plane of the sensory block was detected with a pinprick along the site of surgical incision at 15 and 30 min following the procedure. To confirm the boundary, it was necessary to ensure that the distance between each test point was less than 1 cm. The line between the targeted operative vertebra and the spinous process of the vertebra above was the surgical incision. If the blockage plane totally covered the surgical incision site, it would be recorded as complete blocking. If not, the incision length and vertical diameter of hypoalgesia were measured and the coverage rate was calculated as shown in Figure 1. According to the ultrasound image, the reliability of blocking was classified into three levels and recorded. (Level 0: the needle tip and the diffusion of LA were invisible; Level 1: the needle tip was invisible, the diffusion of LA was visible; Level 2: both the needle tip and the diffusion of LA were visible.) Local anesthetic allergy, toxicity, total spinal anesthesia or epidural block, hematoma, and postoperative infection were recorded. The treatments were also recorded.

Secondary endpoints included sensory decrease in the anterior thigh and quadriceps strength decrease. Pinprick sensation of the bilateral anterior thigh and quadriceps strength were evaluated at 30 min after the completion of the ESP block, and the results were recorded. Decreased quadriceps femoris strength was defined as less than grade 4. All examinations were performed by the same investigator who was unaware of group assignment. Intraoperative and postoperative data were recorded. The TCI concentration was recorded at the following timepoints: endotracheal intubation, skin incision, pedicle screw implantation, decompression, and skin closure. The consumption of anesthetics such as propofol, sufentanil, remifentanil, and rocuronium bromide was recorded. Postoperative pain was assessed at 2, 4, 8, 12, and 24 h using VAS scores of pain at rest and active movement. Moving from the supine to the lateral position was defined as active movement. Sufentanil consumption was recorded at the above-mentioned timepoints. Postoperative nausea and vomiting (PONV) and rescue analgesia were recorded.

### Statistical analysis

#### Sample size determination

The primary purpose of the study was to evaluate the rate of complete coverage of the surgical incision by the sensory block after the completion of a single-level or two-level ESP block. On the basis of a pilot study of 10 patients, the rate after the one-level ESP block was 80%. Assuming that the rate after the two-level ESP block was 100%, the number of patients required for each group was determined as 39, using PASS 11.0 software (NCSS, LLC. Kaysville, Utah, USA) on “Tests for two proportions [proportions]” with 90% power and 0.05 alpha error.

#### Outcome analysis

Statistical analysis was performed on SPSS software Version 22.0 (IBM Corp, Armonk, NY, United States). Continuous variables data were expressed as mean ± standard deviation (SD) if the measurement data were in line with normal distribution. If not, it would be presented as median (interquartile). Statistics of data normality test was performed for continuous variables. Distributed data comprising continuous variables were analyzed using Student’s *t*-test, otherwise, the Mann–Whitney *U* test was used. Categorical data were analyzed using the *χ*^2^ test. If the expected value was less than 5, the Fisher's Exact Test was used. A value of *P* < 0.05 was considered statistically significant.

## Result

**Figure 2:**
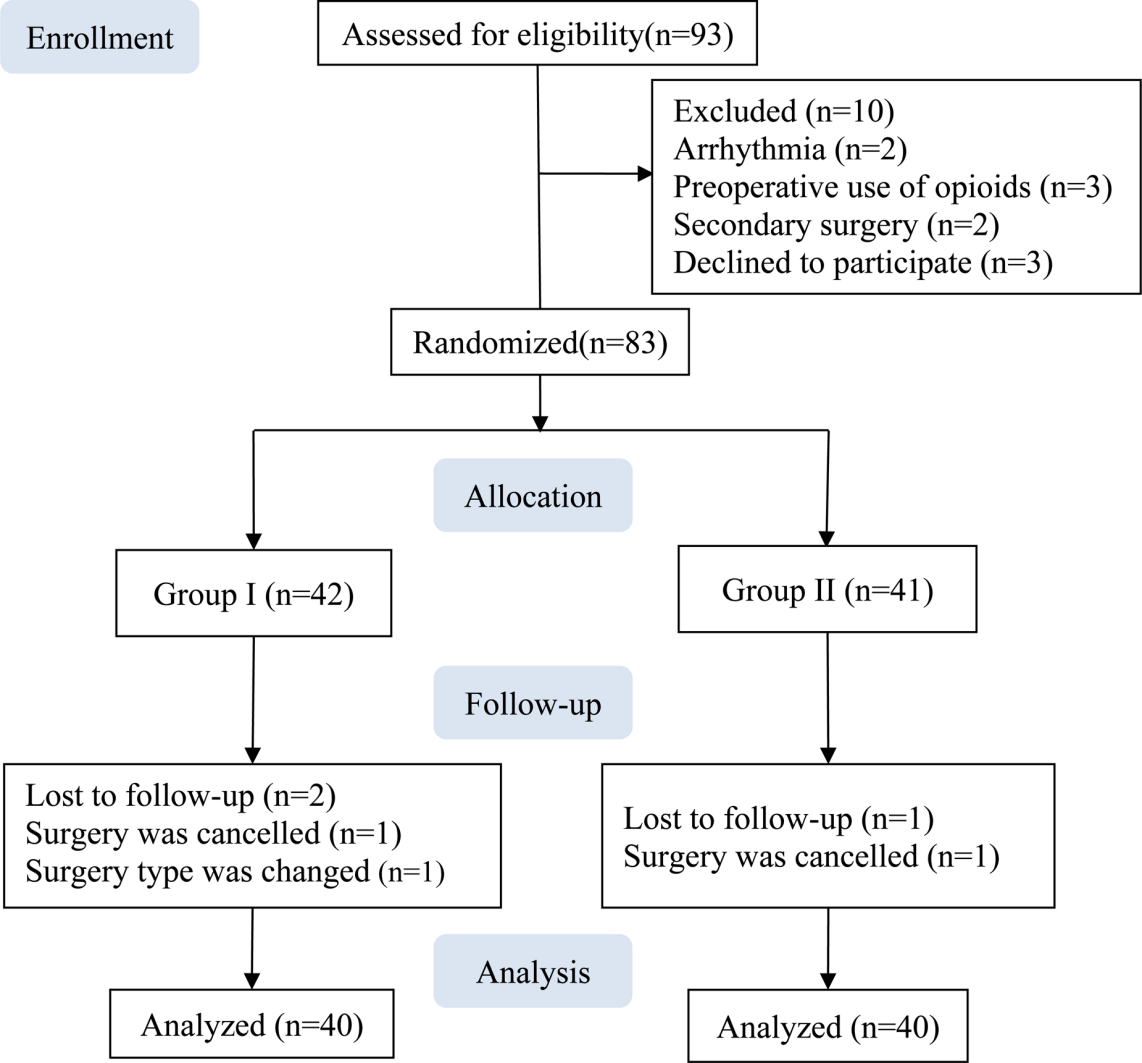
Study flow diagram.

A total of 83 patients were enrolled between March 2021 and August 2021. Two patients from Group I and one patient from Group II were excluded owing to a change in the surgery type or cancelation, and data from the remaining 80 patients were included in the analysis, with 40 in each group (Figure 2).

Demographic characteristics are presented in Table 1. There was no significant difference in terms of sex, age, height, weight, ASA classification, and duration of surgery between the groups.

**Table 1:** Demographic and operative characteristics of the study patients.

	Group I (*n* = 40)	Group II (*n* = 40)	*P*-value
Sex (M/F)^a^	11/29	15/25	0.340
Age (year)^b^	59.1 ± 8.6	59.8 ± 8.6	0.737
Height (cm)^b^	164.5 ± 6.4	163.0 ± 5.4	0.279
Weight (kg)^b^	68.3 ± 8.4	68.8 ± 9.1	0.800
ASA status (I/II)^a^	23/17	19/21	0.370
Duration of surgery (min)^c^	120 (110–120)	120 (103–120)	0.505

Data are presented as mean ± SD or median (interquartile range).

^a^ *χ*^2^ test was used.

^b^ Student’s *t-*test was used.

^c^ Mann–Whitney *U* test was used.

At 30 min after the ESP block procedure, all patients in Group II received a complete sensory block over the surgical incision site. The rate of the complete coverage of the surgical incision by the sensory block was significantly higher in Group II than in Group I (100% [40/40] vs. 85.0% [34/40], *P *= 0.026). Six patients in Group I did not receive complete coverage as described (III) in Figure 1, and the coverage rates were 72.9%, 83.2%, 87.3%, 83.9%, 88. 4%, and 91.4%. At 30 min after the completion of the block, hypoesthesia and muscle strength were assessed for both the left and the right lower limbs, and 80 evaluations were performed in each group. The rate of the sensory block in the anterior thigh was higher in Group II (43.8% [35/80] vs. 27.5% [22/80], *P *= 0.032), but the rate of quadriceps strength decrease did not differ significantly between the groups. The time to complete the ESP block was significantly longer in Group II than in Group I (16.0 [14.3, 17.0] min vs. 9.0 [8.3, 9.0] min, *P *= 0.000). After 15 min of the ESP block procedure, the rate of coverage of the surgical incision by the sensory block was significantly higher in Group II than in Group I (80.0% [32/40] vs. 57.5% [23/40], *P *= 0.030). Group I patients received 80 injections and Group II received 160 injections as described above. There was no significant difference with regard to the reliability of blockage and the targeted vertebral of the upper level (Table 2).

**Table 2:** Target vertebra of the block and block effect.

	Group I (*n* = 40)	Group II (*n* = 40)	*P*-value
Objective vertebral of the upper level (T_12_/L_1_/L_2_/L_3_/L_4_)^a^	0/11/18/11/1	1/14/20/5/0	0.364
Objective vertebral of the lower level (/L_4_/L_5_/S_1_)	None	12/15/13	None
Duration of ESP blocking manipulation (min)^b^	9.0 (8.3–9.0)	16.0 (14.3–17.0)*	0.000
Reliability of blockage (0/1/2)^c^	0/45/35	0/94/66	0.712
Coverage rate ≥ 100% at 15 min after ESP blocking^c^	23 (57.5)	32 (80.0)*	0.030
Coverage rate ≥ 100% at 30 min after ESP blocking^a^	34 (85.0)	40 (100.0)*	0.026
Hypoalgesia of lap^c^	22 (27.5)	35 (43.8)*	0.032
Quadriceps strength weakening^c^	17 (21.3)	26 (32.5)	0.108

Data are presented as median (interquartile range) or number (%).

^a^ Fisher's Exact Test was used.

^b^ Mann–Whitney *U* test.

^c^ *χ*^2^ test was used was used.

*P < 0.05 compared with Group I.

The targeted infusion concentration of remifentanil in Group II was lower than in Group I during intervertebral decompression (2.9 ± 0.3 ng/ml vs. 3.3 ± 0.5 ng/ml, *P *= 0.007). No significant difference was found between the two groups with regard to the target concentration of propofol and remifentanil during the operation (Table 3). There was also no significant difference with regard to the intraoperative anesthetic dosage between the two groups (Table 4). Postoperative rest and active movement VAS scores within 24 h are given in Table 5. There was no significant difference in the total consumption dosage of sufentanil within 24 h postoperatively (Table 6).

**Table 3:** Comparison of the TCI concentration of propofol and remifentanil during the maintenance of anesthesia.

	Group I (*n* = 40)	Group II (*n* = 40)	*P*-value
Propofol (µg/ml)			
Intubation	3.1 ± 0.4	3.1 ± 0.4	0.873
Skin incision	3.6 ± 0.4	3.6 ± 0.5	0.651
Pedicle screw implantation	3.7 ± 0.4	3.8 ± 0.5	0.219
Decompression	3.7 ± 0.4	3.7 ± 0.5	0.581
Skin closure	3.7 ± 0.5	3.8 ± 0.5	0.828
Remifentanil (ng/ml)			
Intubation	1.6 ± 0.2	1.5 ± 0.2	0.726
Skin incision	2.8 ± 0.7	2.7 ± 0.6	0.985
Pedicle screw implantation	2.9 ± 0.7	2.8 ± 0.5	0.390
Decompression	3.3 ± 0.5	2.9 ± 0.3*	0.007
Skin closure	2.7 ± 0.6	2.8 ± 0.5	0.923

Data are presented as mean ± SD.

Mann–Whitney *U* test was used.

*P < 0.05 compared with Group I.

**Table 4:** Comparison of anesthetics consumption dosage between the two groups during the maintenance of anesthesia.

	Group I (*n* = 40)	Group II (*n* = 40)	*P*-value
Propofol (mg)^a^	1041.3 ± 185.4	1057.0 ± 136.5	0.667
Sufentanil (μg)^b^	22 (21–22)	22 (21–23)	0.682
Remifentanil (μg)^a^	861.2 ± 142.3	898.3 ± 128.4	0.225
Rocuronium Bromide (μg)^b^	40 (40–50)	40 (40–48)	0.278

Data are presented as mean ± SD or median (interquartile range).

^a^ Student’s *t*-test was used.

^b^ Mann–Whitney *U* test was used.

**Table 5:** Comparison of visual analog pain scores at postoperative timepoints.

	Group I (*n* = 40)	Group II (*n* = 40)	*P-*value
At rest			
2 h	1.6 ± 1.1	1.6 ± 1.0	0.884
4 h	1.6 ± 1.0	1.5 ± 1.0	0.831
8 h	1.5 ± 0.8	1.3 ± 1.9	0.559
12 h	1.7 ± 0.8	1.6 ± 0.9	0.813
24 h	1.5 ± 0.8	1.4 ± 0.7	0.473
During active movement			
2 h	2.5 ± 0.9	2.3 ± 0.9	0.577
4 h	2.7 ± 0.7	2.7 ± 0.9	0.875
8 h	2.6 ± 0.7	2.6 ± 0.9	0.925
12 h	2.9 ± 0.6	2.7 ± 0.7	0.132
24 h	2.7 ± 0.7	2.8 ± 0.9	0.587

Data are presented as mean ± SD.

Mann–Whitney *U* test was used.

**Table 6:** Comparison of sufentanil consumption dosage in the first 24 h following surgery.

	Group I (*n* = 40)	Group II (*n* = 40)	*P*-value
0–2 h (µg)	4.4 ± 1.9	4.5 ± 2.1	0.987
2–4 h (µg)	4.4 ± 2.1	4.8 ± 2.4	0.483
4–8 h (µg)	8.9 ± 2.2	8.4 ± 2.0	0.304
8–12 h (µg)	10.4 ± 2.9	9.8 ± 2.5	0.382
12–24 h (µg)	22.6 ± 3.3	21.8 ± 2.4	0.411
Total 24 h (µg)	50.7 ± 6.5	49.3 ± 7.0	0.273

Data are presented as mean ± SD.

Mann–Whitney *U* test was used.

No block failures, local anesthetic allergy, toxicity, total spinal anesthesia or epidural blocking, hematoma, and postoperative infection were detected. There was no difference with regard to rescue analgesia, nausea, and vomiting among these patients (Table 7).

**Table 7:** Comparison of rescue analgesia and PONV between the two groups.

	Group I (*n* = 40)	Group II (*n* = 40)	*P*-value
Rescue analgesia	2 (5.0)	1 (2.5)	0.500
PONV	3 (7.5)	4 (10)	0.500

Data are presented as number (%).

Fisher's Exact Test was used. PONV, postoperative nausea and vomiting.

## Discussion

Skin, muscle, and bone will be damaged during posterior lumbar surgery (11). The surgical incision and paravertebral muscles were innervated by the dorsal rami of the spinal nerves, which runs downward and backward after passing through the transverse process of the lower vertebrae. A segmental and crossed distribution is the feature of the dorsal spinal nerve rami. An ideal regional block should block several dorsal ramies of the spinal nerve, especially the nerve from the cranial vertebrae, so we chose one level above the operative vertebrae as the site of the ESP block (9, 15–17). Previous studies have shown that a median of 5 ml of injectate was needed to cover one vertebral level. When the ESP block was performed in the lumbar region in our study, 0.3% ropivacaine of 25 ml was injected (18). In the pilot study, we evaluated the pain sensorial blockage after the ESP block, which did not cover the lower part of the incision. A larger volume of LA might result in a broader block site, but it might cause epidural anesthesia (19). On the other hand, the lumbar ESP block has a more localized spread compared with the thoracic ESP block because of a more complex, multilayered thoracolumbar fascia and the arrangement and thickness of the lumbar musculature (20, 21). The iliolumbar ligament, which passes from the tip of the transverse process of the L5 vertebra to the iliac crest, forms a thickened lower border of the two layers of the TLF and limits caudal spread (22, 23). Because of the reasons cited above, we performed a two-level ESP block, rather than increasing the volume of LA. Different LA-injected levels might result in different blocking sites. We recorded the injected level of the upper ESP block. Because the injected upper level was the same between the two groups, the added lower-level injection site was the reason for better coverage. To make sure LA was injected correctly, the reliability of the block was evaluated. The diffusion of LA was visible on ultrasound image. The result showed that the two-level ESP block provided a better pain sensorial blockage. Similar to our study, Silnha et al. (24) found that the two-level ESP block resulted in a better cranio-caudal spread of LA in a patient undergoing kyphosis correction surgery.

The spread of LA after the ESP block may follow different pathways, such as between the transverse process and the erector spinae muscle, between the QL muscle and the psoas muscle, and between the QL and the erector spinae muscle, which could block both the ventral and the dorsal rami of the spinal nerve (25). Previous studies have shown that different LA volumes, block levels, and needle tip positions lead to different sensory block areas (10, 18, 22, 26–28). In a cadaveric study, 20 ml of contrast solution was injected at L4, and then CT scan and dissection were performed. It was found that the solution spread from L2 to L5 in the erector spinae muscle, reaching the facet joints and the thoracolumbar fascia. In 33% of patients, the solution did not spread anterior to the transverse process, and in 16% patients, the contrast solution reached the corresponding spinal nerves (28). Harbell et al. (22) found that 20 ml of methylene blue injected at L4 could consistently spread to the dorsal rami, but there was no anterior spread to the ventral rami or paravertebral space. Azevedo et al. (27) performed the ESP block at L4 in fresh cadavers, injecting different volumes of LA, and found that the lumbar ESP block was effective in reaching the dorsal rami of the lumbar spinal nerves with a low volume injection of 20 ml. However, the anterior spread reaching the ventral rami or paravertebral space was better achieved with larger volumes of solution (30–40 ml). In our study, an LA of 35 ml was injected for patients in Group II, which yielded a higher rate of anterior thigh analgesia, indicating that LA had spread to the ventral rami. The lumbar disc is innervated by the anterior rami and the sinusoidal vertebral nerve. Better ventral rami blocking might account for a lower target infusion concentration of remifentanil needed in Group II patients during intraoperative decompression.

Multimodal analgesia was used in our study to relieve postoperative pain. In Group I, there were six patients who did not reach 100% incision blockage with the ESP block, but the coverage rate was more than 70%, which could be considered effective for intraoperative and postoperative pain control. There was no difference in terms of the degree of postoperative pain and sufentanil dosage between the two groups, which might be attributed to effective administration of incisional analgesia, and the result may also be limited by the sample size.

This study has some limitations. First, for patients in Group II, a larger volume of LA was injected, and it might result in a wider site of the block. The added injected position in Group II was another factor that might lead to better coverage. However, we could not distinguish whether it was the larger volume, LA-injected position, or both that led to the increased blockage rate. In addition, in our study, the injection point of Group I patients was similar to the upper injection point of Group II. For the one-level ESP block, injection in the midpoint of the incision might result in better coverage. Further study is needed to confirm this result. Second, the dressing covering the incision after surgery made it difficult to evaluate sensory loss of the block, and therefore, the duration of the block was not evaluated. Third, the patients in this study could not be blinded to the intervention, and this might lead to additional bias.

In conclusion, when compared with the one-level ESP block, the two-level ESP block with a larger-volume LA provided better craniocaudal spread and a higher rate of complete coverage of the surgical incision by the sensory block. However, there is no difference in intraoperative and postoperative opioid-sparing effects between the one-level and the two-level ESP blocks. The optimal method of the ESP block in patients undergoing lumbar surgery remains to be explored.

## Data Availability

The original contributions presented in the study are included in the article further inquiries can be directed to the corresponding author.
